# Modelling the Structure and Dynamics of Biological Pathways

**DOI:** 10.1371/journal.pbio.1002530

**Published:** 2016-08-10

**Authors:** Laura O’Hara, Alessandra Livigni, Thanos Theo, Benjamin Boyer, Tim Angus, Derek Wright, Sz-Hau Chen, Sobia Raza, Mark W. Barnett, Paul Digard, Lee B. Smith, Tom C. Freeman

**Affiliations:** 1 The Roslin Institute and Royal (Dick) School of Veterinary Studies, University of Edinburgh, Easter Bush, Edinburgh, Midlothian, Scotland, United Kingdom; 2 MRC Centre for Reproductive Health, University of Edinburgh, The Queen’s Medical Research Institute, Edinburgh, United Kingdom

## Abstract

There is a need for formalised diagrams that both summarise current biological pathway knowledge and support modelling approaches that explain and predict their behaviour. Here, we present a new, freely available modelling framework that includes a biologist-friendly pathway modelling language (mEPN), a simple but sophisticated method to support model parameterisation using available biological information; a stochastic flow algorithm that simulates the dynamics of pathway activity; and a 3-D visualisation engine that aids understanding of the complexities of a system’s dynamics. We present example pathway models that illustrate of the power of approach to depict a diverse range of systems.

## Abstract 2

This Community Page presents a biologist-friendly method for compiling detailed graphical models of biological pathways from the literature, including their subsequent parameterization for use in dynamic simulations of their activity.

This Community Page is part of the Cool Tools Series.

## How to Draw Diagrams of Biological Pathways?

Biologists the world over love a good diagram. The old adage of a picture being worth a thousand words holds true. Diagrams continue to form the bedrock of our efforts to communicate ideas about the complexity of biological systems and pathways. They act as pictorial reviews of what is known about the components of a given system and how they interact. However, there is a fundamental problem with most biological pathway diagrams: they are produced on an ad hoc basis with no rules governing their composition or content. As a result, they convey information only in the narrow context of the accompanying legend, text, or talk, and as a collective resource are of limited use as they cannot be directly compared or integrated.

If pathway modelling is to be more widely adopted by biologists and the results of their efforts useable by others, a new approach is required. Here, we describe a modelling framework that consists of a formalised graphical language that supports the depiction of complex biological interactions and molecules, allowing the creation of detailed models of events based on known information. Once parameterised, these models can then be used directly for experiments that seek to simulate their activity under a variety of conditions. The approach utilises flexible and simple-to-use tools that make diagrams easy to draw and edit as new knowledge arises, and to model their behaviour. Most importantly, the resulting pathway diagrams are useful in the communication of ideas, in hypothesis testing, in enhancing the understanding of the dynamic characteristics of the system modelled, and thereby in helping to drive forward biological research objectives.

The post-genomic era has led to the generation of a massive amount of data on the interactions between molecules across cell types and disease processes. Pathguide.org [1] lists and categorises many of the available resources providing access to this information; it currently lists 547 websites and databases (as of May 2016). Many are databases of collated biological interaction data, experimentally determined or inferred; some well-known examples include String [2], GeneMania [3], ConsensusPathDB [4], IntAct [5] and BioGrid [6]. Other initiatives have sought to ensure there are common standards to exchange this information such as Pathway Commons [7] and PSICQUIC [8]. However, these resources store and serve up interaction data primarily as pairwise interactions, in which the context, nature, or result of these interactions are generally not shown. In other words, the data exist as a simple network of interactions, not as a formalised pathway diagram. Representing the interactions between biological components as pathways is a challenge, and a number of notation schemes have been proposed [9–15], culminating in the establishment of the Systems Biology Graphical Notation (SBGN) project. In 2009, the SBGN community proposed a number of standards for pathway depiction including the “process description language” [12] based on ideas first proposed by Kitano et al. [9]. In process description diagrams, components of a pathway are depicted using a standard set of shapes (glyphs), and both the nature of the interactions between components and the products of those interactions must be shown explicitly. Since its original description, a number of individual models have been constructed based on this approach [16–22], and a number of databases [23–26] now provide centralised pathway resources constructed using a SBGN-like process description notation. Other popular pathway diagram databases such as KEGG [27] and WikiPathways [28]use their own notation systems. Whilst adoption of SBGN’s process description language by parts of the community undoubtedly represents a step in the right direction, many aspects of the SBGN scheme are limiting; the depiction of many common concepts in biology are not supported and the computer-science–based language used to describe it can be alienating to biologists. Finally, tools that support SBGN pathway construction are not always easy to use or have limited functionality and, at present, models cannot be used directly for activity simulation experiments.

## A Biologist-Friendly Approach to Pathway Modelling

**Fig 1:**
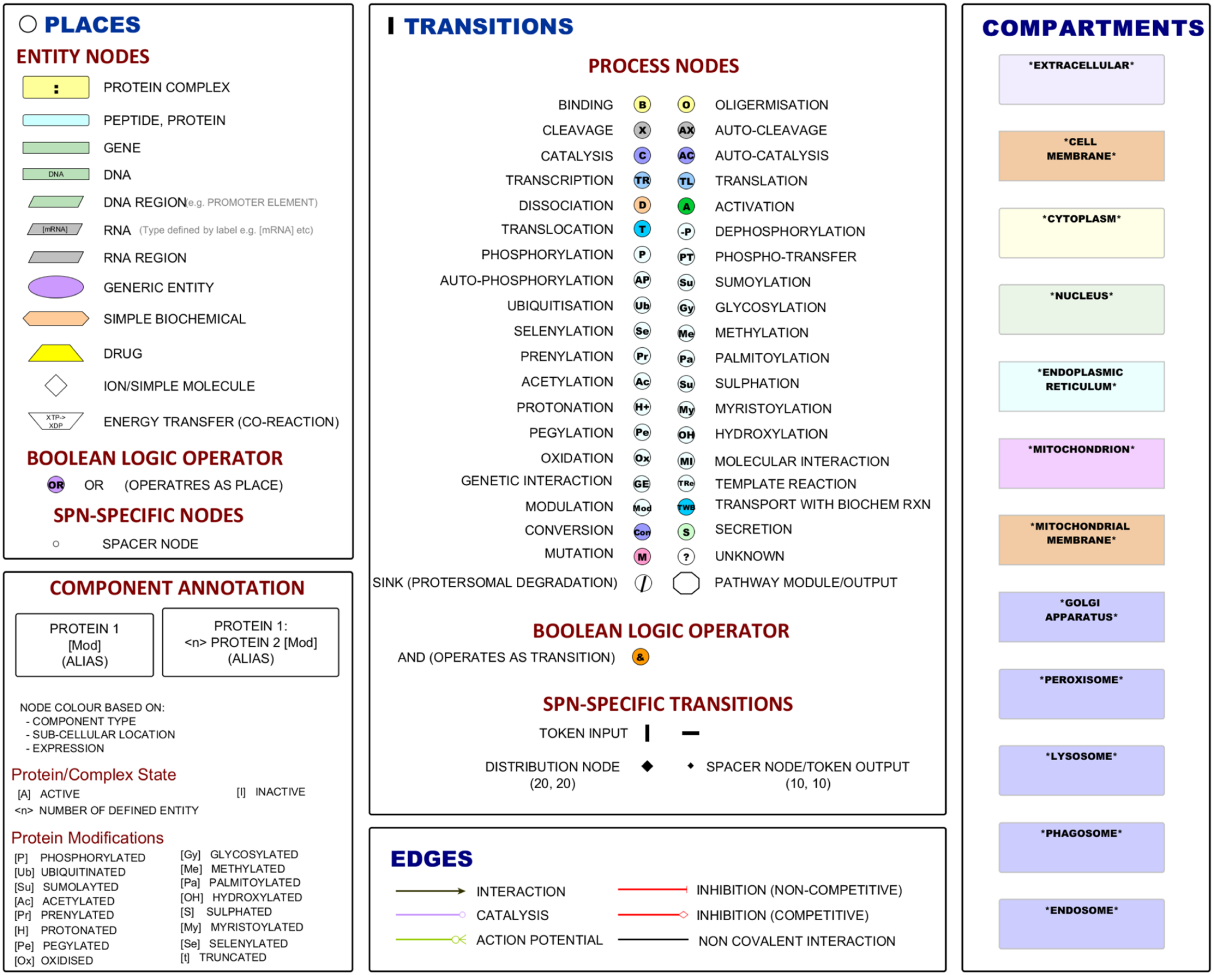
The modified Edinburgh Pathway Notation (mEPN) scheme, version 2.0. Nodes are classed as being either a “place” or “transition” and are connected by “edges.” The scheme consists of 12 different entity nodes (top left panel) representing different kinds of molecular species. Also included are a Boolean logic “OR” operator node and a spacer node that may be required to maintain bipartite structure. Entity nodes are annotated to describe the identity and state of the entity, e.g., protein symbol and its modifications (bottom left panel). Also included in the scheme are 45 different process nodes (top central panel), the majority representing different interactions that can take place between place pathway entity nodes. Also included in this class of nodes are a Boolean logic “AND” operator node, a sink node, two token input nodes, and a spacer node. There are six possible connecting edges (bottom central panel) that represent the nature of the interaction between a place and a transition, two of which are inhibitor edges and one of which is a non-directional edge that represents a non-covalent interaction. Components are represented in the context of their subcellular location, represented by “container” nodes of specific colours (right panel). In all cases, colour is not essential to defining node type but is designed to aid visual distinction between them.

A number of years ago, we started to explore the problem of how does one draw a “good” diagram of a biological pathway. In order to ensure the usability and widest appeal of our approach, we have strived to ensure that the diagrams (graphical models) produced were able to depict events as described in the literature in as much detail as possible whilst maintaining their readability. We also wanted the approach to be understood and useable by relatively junior biologists, as in many cases they were the ones constructing models. To these ends, we developed the modified Edinburgh Pathway Notation (mEPN) scheme (for an up-to-date version, see Fig 1). In common with SBGN, the mEPN scheme is also based on the principles of the process diagram (see Box 1 for a comparison of the mEPN scheme to SBGN). The mEPN scheme is designed to be clear and concise, and to embrace the use of colour to enhance the readability of diagrams. The excellent yEd (www.yWorks.com) software is the primary pathway drawing tool, providing a platform that is robust, flexible, and free to use. It has a range of features that allows a user to link parts of the model to external resources or other supporting data (e.g., PDF files of informative papers and websites) and provides an easy-to-use platform for diagram visualisation and editing. An extended description of construction of mEPN models is provided in S1 Text, and a protocols paper in which we have sought to provide a detailed guide to any would-be pathway modeller is available on bioRxiv preprint server (http://dx.doi.org/10.1101/047043).

**Box 1. mEPN Versus SBGN**

**Fig 2:**
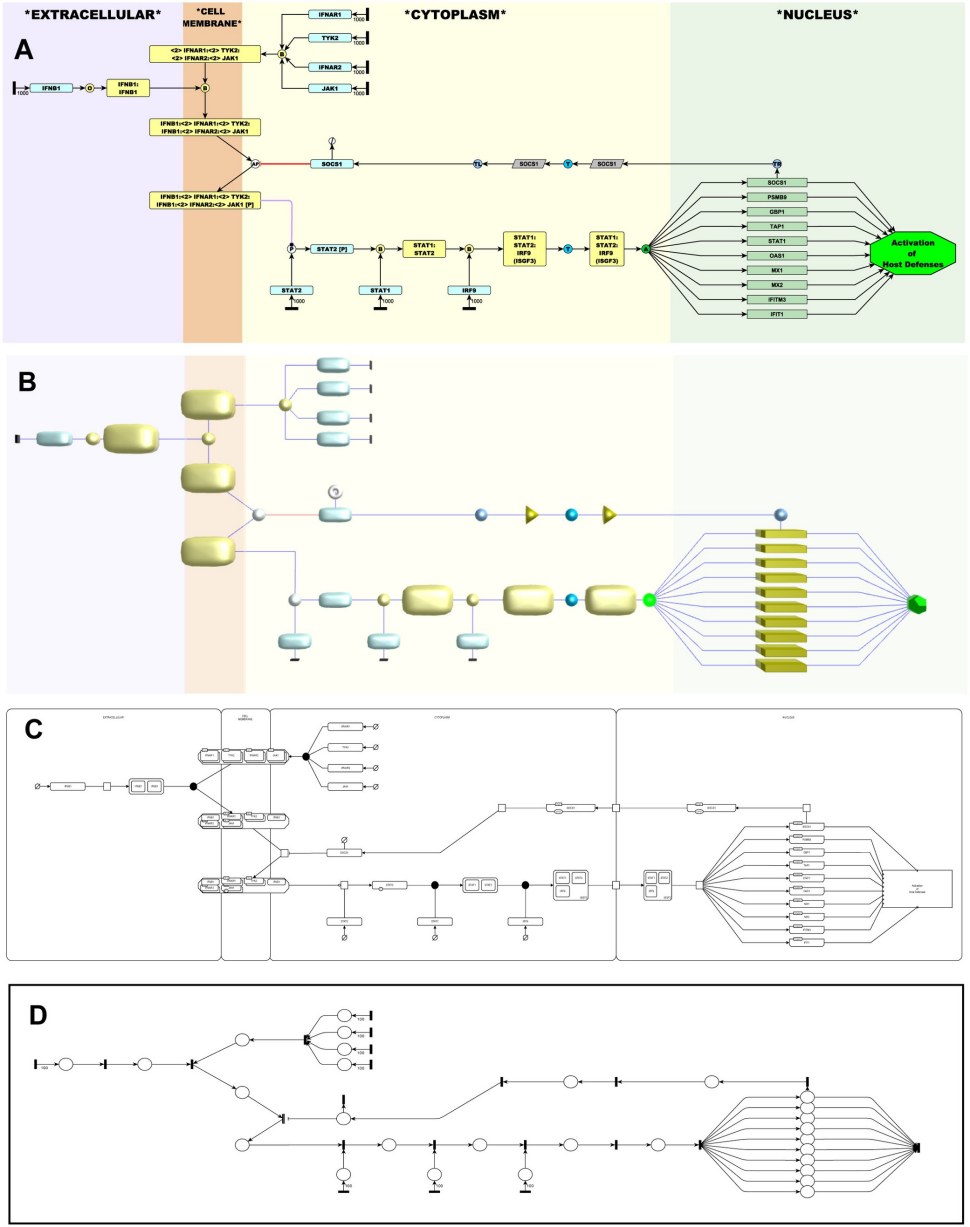
A simple pathway represented in four different ways. This figure represents a simplified model of the interferon-β signaling pathway. (A) Shows this pathway represented using the mEPN scheme and parameterised ready to run a simulation, (B) when imported into BioLayout *Express*^3D^ and visualised using the mEPN 3D notation scheme, (C) as exported from BioLayout as an SBGN-ML file and loaded in the Vanted software, and (D) the same set of interactions represented as a Petri net, illustrating the similarity to mEPN diagrams.

SBGN is perhaps the most widely employed graphical notation scheme in use today for pathway modelling. The objectives of SBGN are aligned with our own, and the mEPN scheme has borrowed concepts from it. Indeed, models constructed using the mEPN scheme may be exported from within BioLayout *Express*^3D^ as a SBGN-ML file, from whence they may be viewed and edited within tools that support SBGN, e.g., Vanted (Fig 2) [29]. In our experience, however, the SBGN scheme is overly complicated for an audience of biologists and is limiting in terms of its ability to depict certain processes; we believe the mEPN scheme offers certain advantages. However, where possible, we have attempted to maintain consistency with the SBGN standard, but there are a number of areas where we have chosen to deviate from it.

**Fig 3:**
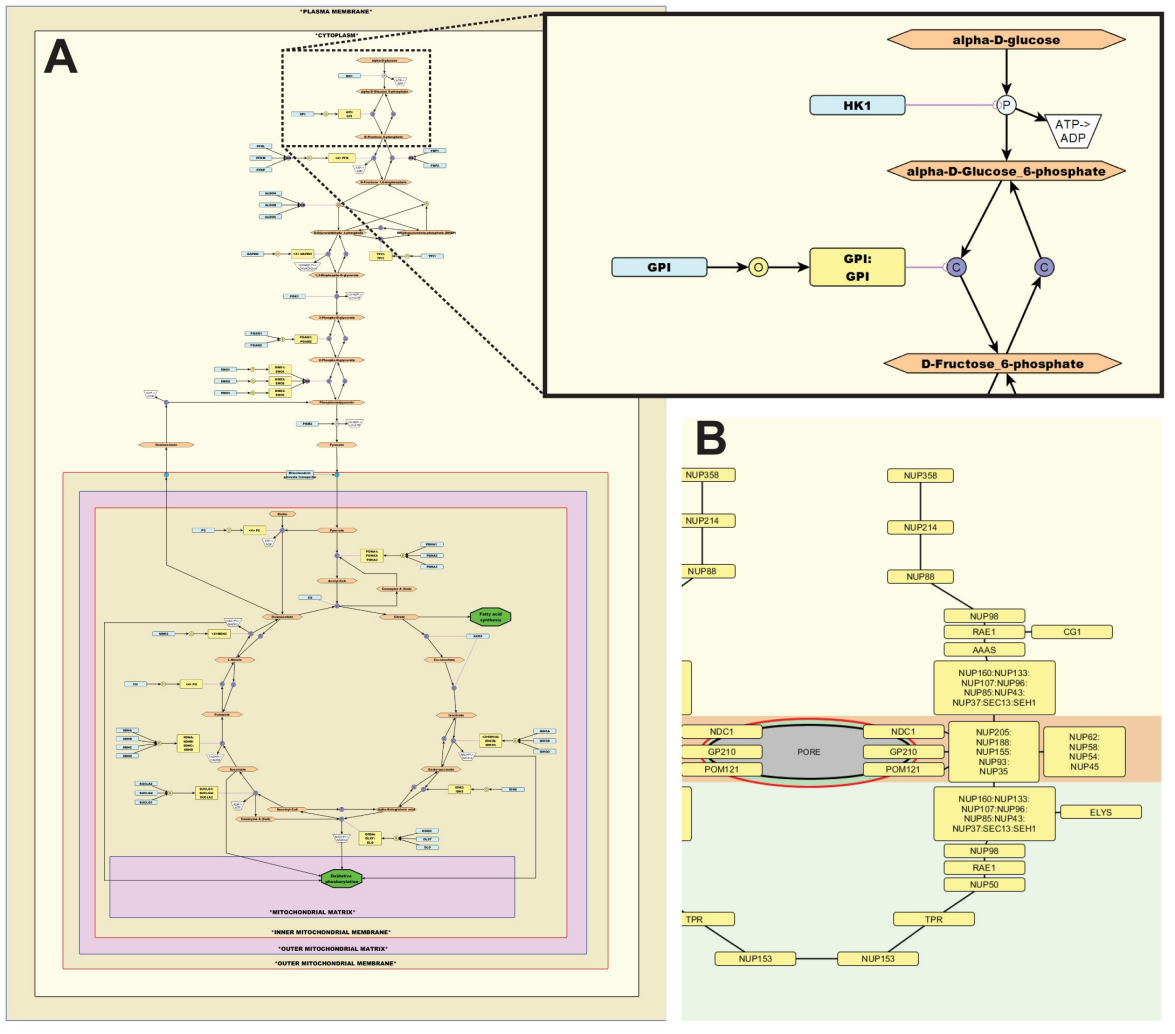
Examples of pathways and complexes represented using mEPN. (A) A model of the human TCA cycle and glycolysis pathways drawn using the mEPN scheme. The insert shows a small section of the pathway demonstrating the different shapes and colours used to depict different classes of molecule and how different types of processes can be distinguished using differently labelled process nodes. This is a purely graphical model, but with parameterisation it could be used to run simulations of this pathway. (B) Depiction of the nuclear pore complex as an example of how the mEPN scheme can be used to represent large structures composed of multiple subunits. In all models constructed by us, standard nomenclature is used to define entities (e.g., Human Genome Nomenclature Committee [HGNC]-approved names for human proteins and genes) to remove ambiguity of what is being represented.

1. SBGN uses a number of non-geometric shapes to depict a number of pathway entity types (or EntityPoolNodes, as they are referred to in SBGN). These shapes are not supported by yEd or, indeed, other generic network editors and are not used in the mEPN scheme.
2. Protein complexes are represented in SBGN using either a “stack of proteins” glyph in the case of homo-multimers or as a series of individual protein subunit glyphs within container node in the SBGN scheme. In mEPN, all such entities are represented as a single rounded rectangle glyph in which the number and name of constituent entity nodes are defined by the node’s label; we see no advantage graphically or otherwise in rendering complexes by the means proposed by SBGN. In drawing larger complexes, we have also often found it useful to show individual proteins/subunits as individual entities joined by edges with no arrows (the so-called “non-covalent interaction” edge) so that the structure and relative position of individual subunits might be better communicated graphically (see Fig 3B, a model of the nuclear pore). However, such models cannot currently be used in the computation modelling environment as they do not conform to requirements for a bipartite graph structure of computational models.
3. SBGN has specific glyphs for association (binding) and dissociation, but almost all other processes are represented by a square box. This gives no information on the process they represent and is an important omission. In mEPN, we currently list 40 types of processes that one might wish to define when constructing a pathway, including all of those associated with the BioPAX level 3 pathway exchange language [30]. Whilst in the computational modelling environment of the signalling Petri Net (SPN), all transition nodes function the same; it is important to differentiate between what are often quite different processes in order to aid understanding and readability of the models. We have added the black rectangle and diamond transitions for modelling purposes.
4. We have simplified the list of edges representing interactions between entity nodes down to interaction, catalysis, inhibition, and the special use case action potential, and do not recognise many of the options available listed by SBGN (i.e., consumption, production, reversible modulation, necessary stimulation, logic, and equivalence arcs), as we have found them difficult to explain to biologists and not necessary to the depiction of pathways.
5. Finally, perhaps the most important issue for computational modelling purposes is that in the current version of mEPN, we define all nodes as being either places or transitions, a division that makes biological sense in terms of what they represent and conforms to the requirement of Petri net models.

An example of a simple model of the signalling pathway of Interferon-β (IFNB1) represented in the mEPN scheme, BioLayout *Express*^3D^, and SBGN diagram or a Petri net can is shown in Fig 2 for comparison.

At its most basic level, a pathway diagram constructed using the mEPN scheme is a simple network composed of nodes and edges. Nodes fall into one of two categories representing either pathway entities or processes. Entity nodes generally represent biomolecules (e.g., RNAs, proteins, complexes, biochemicals), and each class of entity is represented by a glyph of a particular shape. Process nodes represent an event occurring to or between entities (e.g., translocation, binding, dissociation, phosphorylation), and are generally represented by a circle with a two- to three-letter label distinguishing between the different types of processes they represent. The lines that join nodes, called edges, define the direction of an interaction and in some cases the nature of it (e.g., catalysis, action potential, inhibition, or non-covalent interaction). A model of the well-known pathways of the glycolysis and the tricarboxylic acid (TCA) cycle constructed using mEPN is shown in Fig 3A, and an example of how a large complex such in the nuclear pore can be shown to be made up of multiple connected subunits using the non-covalent edge is shown in Fig 3B (these models are also available to download and edit in S1 GraphML).

## Beyond the Picture

A hallmark of many modern biological investigations are genome-scale analyses, which continue to produce huge amounts of data. These inevitably implicate hundreds or thousands of biological entities as being of interest due to their change in abundance in a given experimental or clinical paradigm. However, interpretation of the meaning of such observations represents one of the greatest challenges in biology today. Gene Ontology (GO) enrichment analyses may indicate that a set of gene or protein “hits” are implicated in a given pathway or process, but what role they play or how they function together is not defined. Similarly, and as mentioned above, protein–protein interaction databases may be used to find out if there is any evidence that a set of proteins share a functional association, but the information returned is generally without context. An alternative approach is to overlay results onto pathway diagrams, thereby indicating which of those represented have been regulated. The researcher can then understand not only which pathways have undergone regulation but which specific elements have been altered, allowing them to hypothesise how a given perturbation might affect a pathway’s activity or downstream events. This method is very useful but limited by the quality and scope of the reference pathway diagram. There are a number of tools that currently support data visualisation in the context of networks and pathways, including CellDesigner [31], NaviCell [25], KEGG Mapper [27], ReactomeFiViz [32], iPath [33], and Medusa [34]. As knowledge of biological interactions grows, there is, therefore, a need to construct better models to support these types of analysis, and we present here our approach to constructing such models.

This brings us to perhaps the most challenging aspect of pathway modelling: that of simulating their activity. To truly comprehend a pathway, one needs not only the pictorial representation or “connectivity map” but also to understand the dynamics of the system under different conditions. Computational and mathematical modelling studies aim to simulate biological systems to explain a pathway’s observed activity. When this is achieved, models can be used to explain or predict a system’s response to perturbations (e.g., a drug treatment, gene knockout, etc.) or to predict missing components. Over the years, numerous computational models have been published for a diverse assortment of biological systems (see the BioModels database for examples [35]). Researchers have used a range of approaches to simulate system dynamics, including ordinary and partial differential equations, qualitative differential equations, stochastic equations, directed graphs, Bayesian and Boolean networks, and rule-based formalisms [36,37], and the Systems Biology Markup Language (SBML) has been developed as an open interchange format for such models. Most equation-based models are described primarily by the maths, and graphical representation of the events being modelled is generally an afterthought. Indeed, whilst mathematical modelling of biological pathways was one of the founding activities of systems biology, currently, pathway depiction and pathway modelling are generally considered to be separate disciplines. The aim of one is draw an accurate representation of known events, the other to understand how a pathway might operate as a dynamic system; the two activities are generally not compatible. However, it is the requirement for experimentally derived rate constants to feed into many of these models, and the significant computational power required to solve a series of equations generally limits equation-based approaches to modelling relatively small and well-characterised systems [38]. These factors have limited the routine use of modelling by biologists who are generally not comfortable with the mathematical modelling techniques employed.

## Modelling Pathway Dynamics Using Activity Flow Simulations

Process diagrams share much in common with Petri nets, a modelling approach that has been used to model many different types of systems, with a growing literature describing their use in modelling biological pathways [39–41]. Petri nets are a rule-based approach founded on the concept of modelling the flow of tokens through a system in which tokens represent the activity (or amount) of a given component. Petri nets are networks made up of two types of node: places and transitions (usually represented by circles or black rectangles, respectively). A strict requirement of Petri nets is that they are constructed as bipartite graphs, i.e., they have a recurrent structure of place–transition–place (or in the case of process diagrams, entity–process–entity). The signalling Petri Net (SPN) algorithm we have employed to dynamically model flow through mEPN diagrams was originally described by Ruths et al. [38], which, after refinement, was incorporated into the network visualisation and analysis software BioLayout *Express*^3D^ (www.biolayout.org). As part of this development, we enabled the import and visualisation of mEPN models and thereby the simulation of activity flow through such models. In order to achieve this end, we modified the mEPN scheme to define all nodes as representing either places (which includes all pathway entities) or transitions (which includes all nodes representing processes). An extended description of the SPN modelling system is provided in S1 Text and in the accompanying protocol paper (http://dx.doi.org/10.1101/047043).

Models need to be parameterised prior to running a simulation, i.e., the initial conditions for the experiment need to be set. With this modelling platform, parameterisation is, in principle, relatively simple to perform and is based on information that may be available to a biologist. The primary parameterisation is the structure of a model itself; this is the major determinant of where tokens can flow to and from, and how long it takes for them to move from place to another. Parameterisation is also carried out through the placement of tokens on entities at the starting points of a network, i.e., on places that have no parents. An entity without tokens, or where token number is set to zero, can be considered not to exist (e.g., a knockout or not expressed). The more tokens placed on an entity node prior to running the model, the more of that entity (or the greater its activity) at the beginning of a simulation. The assignment of tokens to an entity node can be arbitrary or inferred from experimental evidence, such as gene expression or proteomics data, where available.

Models constructed using the mEPN scheme, parameterised and then saved in yEd as GraphML files, can be loaded into the tool BioLayout *Express*^3D^. BioLayout has been designed to recognise the different glyphs used in the mEPN scheme and translates all 2-D mEPN glyphs into their 3-D equivalents, e.g., circles become spheres, rounded rectangles become rounded cuboids (Fig 3A and 3B). The 3-D palette of shapes being far larger allows greater flexibility to distinguish between certain entity types. BioLayout can also export a SBGN-ML version of a mEPN model such that it can be loaded into an SGBN-compliant platform such as SBGN-ED [42] as used here (Fig 2C). Shown in Fig 2D is an illustration of how the concepts of the mEPN language map onto the bipartite graph structure of a Petri net.

When an mEPN model is loaded, BioLayout requests whether a simulation is required. On answering yes, a dialogue is presented (S1 Fig) by which conditions for the SPN simulation can be set, i.e., mode of stochasticity, number of runs and time blocks, etc. A simulation is made up of a series of “time blocks” in which each transition in the model is “fired” exactly once in a random order and tokens are moved from the place(s) upstream of the transition to the downstream place(s). In the default mode, the number of tokens moved is essentially random, i.e., between zero and the maximum number of tokens available. A series of time blocks is referred to as a “run,” and a simulation may involve one or many runs, each run beginning at the same initial token marking. The result of a simulation for a given pathway entity node (place) is the average accumulation of tokens across individual runs and, as such, is associated with a measure of variance. The more runs performed, the more reproducible results will be across separate simulations. It should be noted that even when running simulations on large models over many time blocks composed of hundreds of runs, computational times for the SPN algorithm are normally no more than a few seconds (S1 Fig). Following a simulation run, the activity of the system and individual entities within it is represented by the flow of tokens from one entity node to another over time. One way to view results is through the animation of this token flow, a node’s size and colour being used to represent token accumulation over time. This is a capability enabled within BioLayout, which has an interface that allows the user to control many aspects of this animation (S1 Fig). This functionality provides a powerful visual medium to appreciate token flow in large network diagrams and can be used to troubleshoot the innate challenges associated with model construction. Indeed, the general modus operandi when optimising models is to construct them in yED, test the flow characteristics in BioLayout, and solve issues encountered by going back to the model in yED and then retesting the model. In reality, the movement between the two tools is quick, and model testing and improvement can be a rapid process. See S1 Video for a summary of the modelling process.

## Flow Characteristics through Basic Pathway Motifs

### Linear Flow

**Fig 4:**
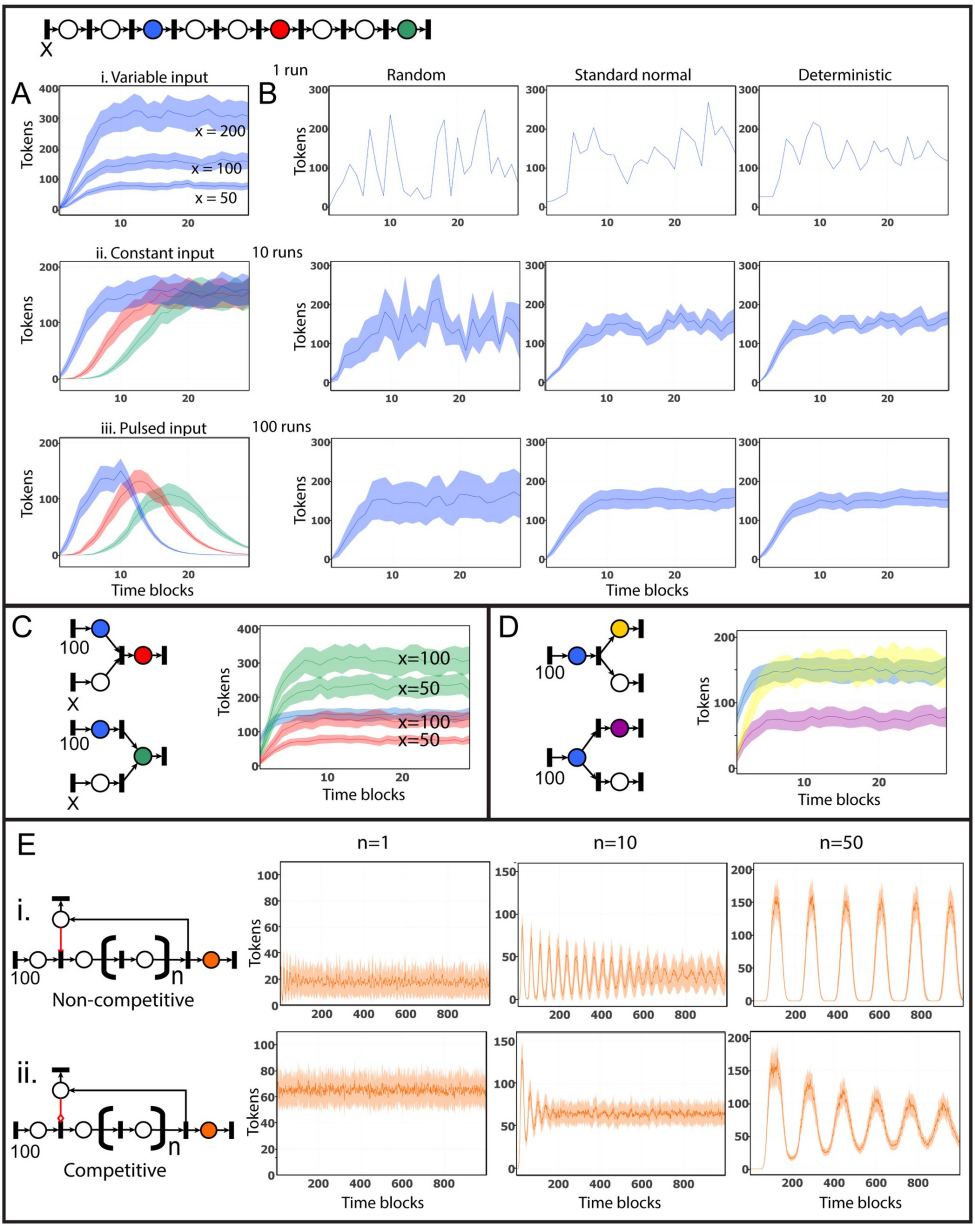
Flow simulation through simple pathway motifs. (A) A linear network with tokens added to the system on the first node (X) illustrates how differences in the placement of tokens and SPN parameters affect token output flow. Token accumulation on the blue, red, and green nodes in the network has been used to illustrate the effect changing the number and/or pattern of tokens introduced. (i) Varying the initial token input (X = 50, 100, or 200), (ii) the rate of token accumulation at different points downstream of a constant input (X = 100), and (iii) token flow following a pulsed input of tokens (100 tokens during time blocks 11–30). (B) Token accumulation on the blue node illustrates the effect on changing the number of runs and stochasticity of the SPN algorithm (X = 100). (C) Modelling multiple inputs into a place. The red node shows two places feeding into a downstream place via a transition node where flow is determined by the upstream node with the least number of tokens. Input to the green node is via separate transitions; token accumulation is additive. (D) Modelling multiple outputs from a place. In the first instance, where output from the blue node is via a single transition, the yellow downstream node receives the same number of tokens as was present on blue, i.e., flow is conserved. When outputs are split from the node itself, token output is distributed equally amongst downstream nodes (purple). (E) Negative feedback loops. In the first case, a non-competitive inhibitor edge (i., red edge with a flat bar) will completely stop token flow through the target transition if any tokens are present on the inhibitor node. By contrast, competitive feedback edge (ii., red edge with an open diamond end), the number of tokens on the inhibitor node are subtracted from the number of tokens flowing through the target transition. An additional factor in determining flow through a negative feedback loop is the number of steps between input and feedback. The more nodes present between feedback receiver and inducer, the more tokens accumulate in the system, resulting in a longer wavelength of the feedback and a greater amplitude of token flow, as illustrated here by the differences between 1, 10, and 50 nodes between signal input and inhibitor. All token flow plots are shown as mean (darker line) with standard error (lighter area around line).

In a linear network consisting of a line of alternating places and transitions, each time a transition is fired, tokens will “flow” down the network; the more tokens added, the more tokens will accumulate downstream (Fig 4A.i). With a constant input of tokens, token accumulation rises to a constant level and remains “steady,” the rate of tokens entering a place approximating those leaving; places close to the start of the line accumulate tokens faster than those further downstream (Fig 4A.ii). When tokens are added in for a fixed number of time blocks (i.e., a pulsed input), a wave of flow is produced, the amplitude of the wave reducing and its wavelength increasing at places downstream of the input (Fig 4A.iii). Variation of flow over time under steady state conditions depends on the number of runs performed and the randomness of signal propagation (Fig 4B).

### Flow through Interactions between Multiple Entities

Pathways are made up of a small number of basic network motifs [43]. When transitions receive numerous inputs, they function as rule-based regulators of token flow. A basic rule is that when the number of tokens on input places is not equal, the number of tokens taken forward will be based on the input place with the least number of tokens (Fig 4C, red). In the case of multiple inputs into places, inputs are additive (Fig 4C, green). In instances where there are multiple outputs from a transition, the number of tokens on the upstream place will be mirrored by downstream places, i.e., flow is preserved (Fig 4D, yellow). Where outputs come direct from a place, the number of tokens on the upstream place will divided randomly amongst downstream places, i.e., flow is divided (Fig 4D, purple).

### Feedback Control

Feedback loops are a fundamental feature of biological systems, and an ability to model them is an essential property of any modelling system. Inhibitor edges have a unique function. They originate from an entity (the inhibitor) and connect with a transition node, i.e., the process that is to be inhibited. This system includes two types of inhibitor edge representing non-competitive and competitive inhibition. The first operates on the basis that if any tokens are present on the inhibitor, flow through the transition is completely blocked. In the second case, the number of tokens residing on the inhibitor is subtracted from the number of tokens flowing through the transition. As no tokens are lost through inhibitor edges, it has become standard practice to draw inhibitor places with an associated output transition (sink). Without this, flow through a negative feedback loop is completely and irrevocably stopped as tokens accumulated on the inhibitor remain there, blocking further flow through the target transition. An inhibitor with a sink transition node attached effectively has a half-life (as tokens are lost from the inhibitor via the sink) and, depending on the configuration of the feedback loop, e.g., path length between input and inhibitor and type of inhibition (amongst other factors), the system may exhibit a range of oscillatory activities (Fig 4E).

**Fig 5:**
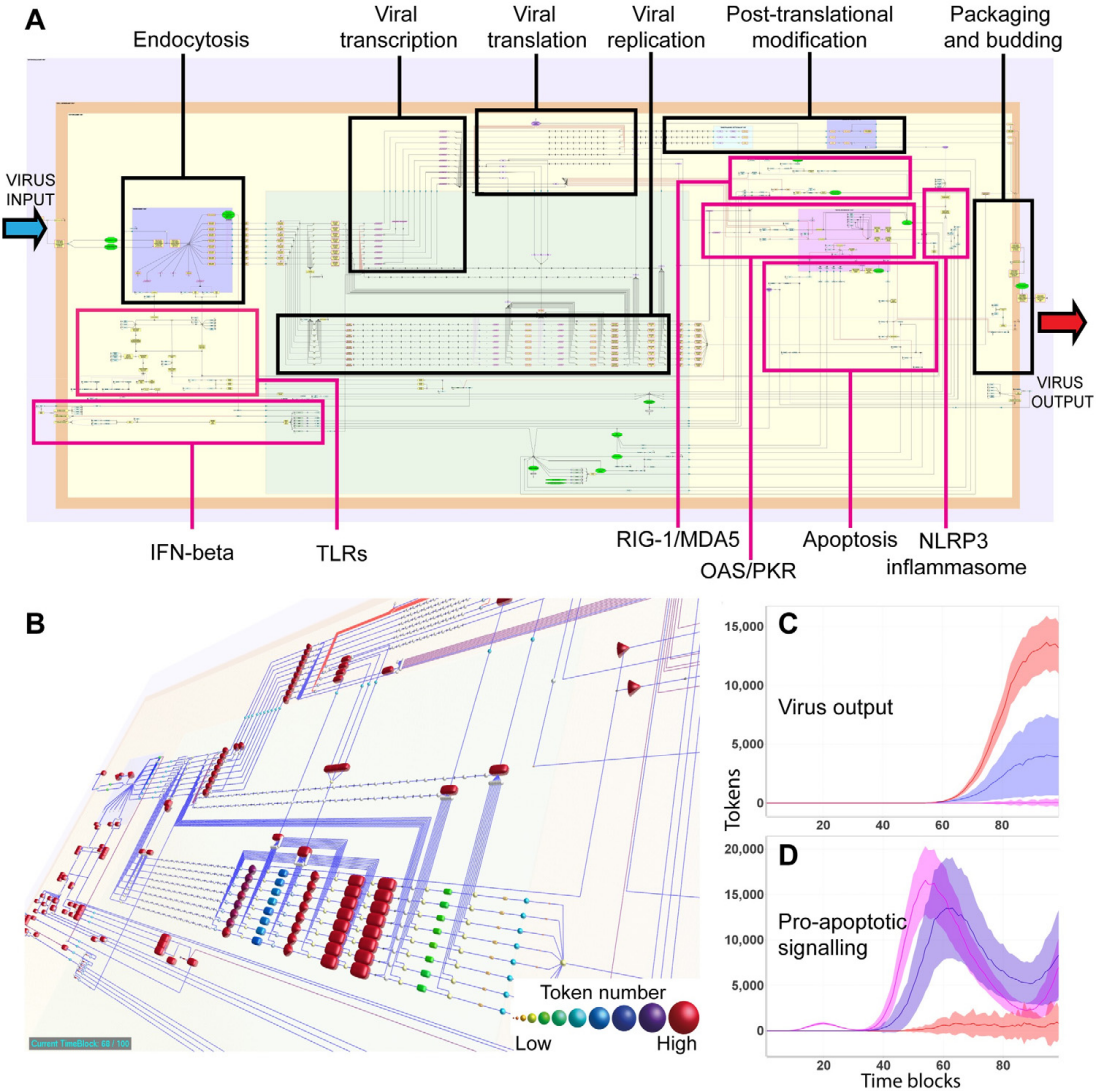
A model of the influenza A life cycle. (A) Representation of a large model of the life cycle of the influenza A virus (IAV) and interactions with the host defence systems in a macrophage. The boxes highlight the modular structure of the model. Modules of the viral replication pathway are indicated with black boxes; modules of the macrophage host defence mechanisms are indicated with pink boxes. Model constructed and visualised in yEd. (B) Visualisation of token flow through the IAV/macrophage model after SPN simulation in BioLayout *Express*^3D^. (C) Approximately 14,000 tokens accumulate on the “virus output” node during SPN simulation of the model when the host defence systems are removed (red) compared to approximately 4,000 for a naïve macrophage (purple) or <10 for an interferon primed macrophage (pink). (D) Pro-apoptotic signalling reaches a peak of approximately 16,000 tokens in interferon-primed macrophages and 14,000 in naïve macrophages, but pro-apoptotic pathways are little activated in the epithelial cell model (<800 tokens). For more details on the assembly and parameterisation of this pathway and to download the pathway itself, see S2 Text, S2 Graphml, S2 Fig and S3 GraphML.

Described above are the characteristics of flow simulations through basic pathway motifs. It should be noted that this method is scalable, and large pathway models consisting of hundreds or possibly thousands of nodes can be constructed and used in flow simulation experiments. We present a recently constructed model of the influenza life cycle in macrophages as an example of such a large-scale model (Fig 5). See also S2 Text, S2 Graphml, S2 Fig and S3 GraphML for a description of this pathway and for access to the model itself.

## The Challenges and Rewards of Pathway Modelling: A Biologist’s Perspective

Described above are the basic characteristics of the flow simulation algorithm and how this approach maps onto pathway diagrams assembled using the mEPN scheme. This interoperability between an advanced graphical notation scheme and a powerful and rapid dynamic modelling platform opens up pathway modelling to a wider audience. However, modelling a pathway of any size is not a trivial undertaking—it takes skill, knowledge, and dedication. From a biologist’s perspective, we would argue that the time and effort required to engage in this activity is rewarded. For most researchers, a current pathway diagram describing their system of interest will not exist, and constructing it themselves can be an extremely useful exercise. It provides an opportunity for a biologist to formalise what they know in a form that can be communicated to their peers or coworkers. It also provides a platform where additional information can be added by others, thereby offering a medium for debate and a route to establishing a common understanding. Indeed, one of the most valuable aspects of pathway modelling is the process of model construction itself and the opportunity it provides to systematically collect and discuss available knowledge. By performing this act, it exposes what is not known or, as is sometimes the case, why events as described could not possibly work the way they are reported to. In a teaching environment, students can be engaged in the activity. They gain valuable experience in learning to mine the literature. They have to learn to be critical of the reliability of information and to judge how to represent information when it is incomplete or conflicting. How they go about this task and what they produce in terms of a “finished” diagram can be judged and marked in the same way as any written assignment.

## Summary

The modelling framework described here is flexible enough to model all the basic network motifs found in all biological pathway types [43,44], allowing curators to represent and capture pathway knowledge in great detail. It also supports the parameterisation of models according to criteria that are readily accessible, i.e., the relative amount of specific entities can often be experimentally determined or inferred, and tokens placed accordingly. Also, parameterisation of models such as introducing amplifications, reductions, or delays to signal propagation can be made simply by marking such events on the models or by altering the structure of the model itself. We present a number of worked examples of pathway models drawn using the mEPN scheme on the *Virtually* Immune website (www.virtuallyimmune.org/) and BioLayout *Express*^3D^ as a means to model their dynamics. In these respects, we believe that the computational framework presented here represents a major advancement in modelling biological pathways. We also envisage that the tools and approaches described here will be useful to other groups: for instance, those investigating single cell biology, where the response of individual cells in a population is heterogeneous and stochastic [45] or synthetic biology, where the properties of an engineered biological pathway module could be tested prior to construction. We also envisage that the methodology will have application in the modelling of complex systems outside of biology.

## Supporting Information

S1 Fig Setting up BioLayout *Express*^3D^ to run and visualise SPN simulations. (A) When an SPN-ready model is loaded in BioLayout *Express*^3D^, the software automatically recognises it and prompts users to run an SPN simulation using this window. The user defines the number of time blocks and runs, whether the variance is displayed, the form of stochasticity with which the model is parameterised, and the transition type. The set-up used for generating visualisations in this article is illustrated. (B) On completion of the SPN simulation run, this window provides a time for the running of the simulation and prompts the user to repeat the simulation, save the SPN results, or open the animation dialog. (C) The animation control dialogue window allows the visualisation of the SPN simulation to be defined. Size and colour of nodes as well as the appearance of token flow and the speed of the animation can be defined here.
(PDF)

S2 Fig Construction and parameterisation of a representation of IAV life cycle in a cell with no host defences. (A) Illustration of the modular structure of the IAV life cycle that facilitates its readability and allows for pathway expansion as new data become available or as a focus of interest grows. (B) When our virtual cell is challenged by a limited amount of virus (MOI 10 for two time blocks, ten virions are represented by ten input tokens, an accumulation of the structural components haemagglutinin (HA), neuraminidase (NA), matrix protein 1 (M1), and nonstructural protein 2 (NS2) both at the (C) mRNA and (D) protein level leading to (E) viral progeny (approximately 1E^4^ virions/cell Virus Output). The model outputs are comparable to the levels of (F) mRNA and (G) protein accumulation seen in previously reported experimental in vitro infection.
(PDF)

S1 GraphML GraphML files of the models shown in Fig 2. (GRAPHML)

S2 GraphML GraphML file of the mEPN pathway representation of IAV life cycle in a cell with no host defences. (GRAPHML)

S3 GraphML GraphML file of the mEPN pathway representation of IAV life cycle in the context of a macrophage (IFNB-primed). (GRAPHML)

S1 Text Methods and an extended description of the SPN modelling system. (DOCX)

S2 Text Description of the assembly and parameterisation of a model of the IAV life cycle in the context of a macrophage. (DOCX)

S1 Video Overview of the mEPN approach to pathway modelling and simulation. (MP4)

## Abbreviations

GO: Gene Ontology
HGNC: Human Genome Nomenclature Committee
IAV: influenza A virus
IFNB1: Interferon-β
mEPN: modified Edinburgh Pathway Notation
SBGN: Systems Biology Graphical Notation
SBML: Systems Biology Markup Language
SPN: signalling (stochastic) Petri Net
TCA: tricarboxylic acid
