# Supplementary material for: Modelling the Structure and Dynamics of Biological Pathways
Source: PLoS Biol. 2016 Aug 10;14(8):e1002530. doi: 10.1371/journal.pbio.1002530 (PMC4980033; doi:10.1371/journal.pbio.1002530)
Supplement: S1 Fig — (A) When an SPN-ready model is loaded in BioLayout Express3D, the software automatically recognises it and prompts users to run an SPN simulation using this window. The user defines the number of time blocks and runs, whether the variance is displayed, the form of stochasticity with which the model is parameterised, and the transition type. The set-up used for generating visualisations in this article is illustrated. (B) On completion of the SPN simulation run, this window provides a time for the running of the simulation and prompts the user to repeat the simulation, save the SPN results, or open the animation dialog. (C) The animation control dialogue window allows the visualisation of the SPN simulation to be defined. Size and colour of nodes as well as the appearance of token flow and the speed of the animation can be defined here. (PDF) [file pbio.1002530.s001.pdf]

A

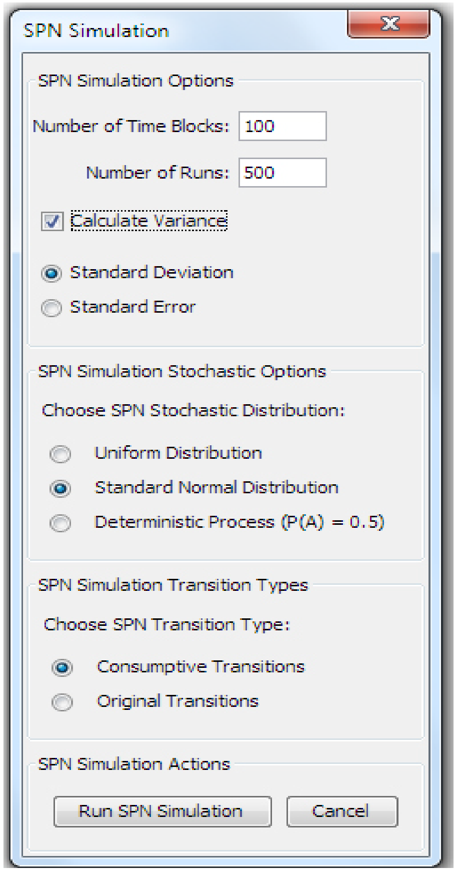
SPN Simulation
SPN Simulation Options
Number of Time Blocks: 100
Number of Runs: 500
Calculate Variance
Standard Deviation
Standard Error
SPN Simulation Stochastic Options
Choose SPN Stochastic Distribution:
Uniform Distribution
Standard Normal Distribution
Deterministic Process (P(A) = 0.5)
SPN Simulation Transition Types
Choose SPN Transition Type:
Consumptive Transitions
Original Transitions
SPN Simulation Actions
Run SPN Simulation
Cancel

B

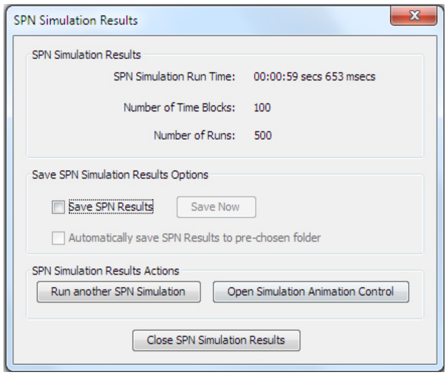
SPN Simulation Results
SPN Simulation Results
SPN Simulation Run Time: 00:00:59 secs 653 msecs
Number of Time Blocks: 100
Number of Runs: 500
Save SPN Simulation Results Options
Save SPN Results
Save Now
Automatically save SPN Results to pre-chosen folder
SPN Simulation Results Actions
Run another SPN Simulation
Open Simulation Animation Control
Close SPN Simulation Results

C

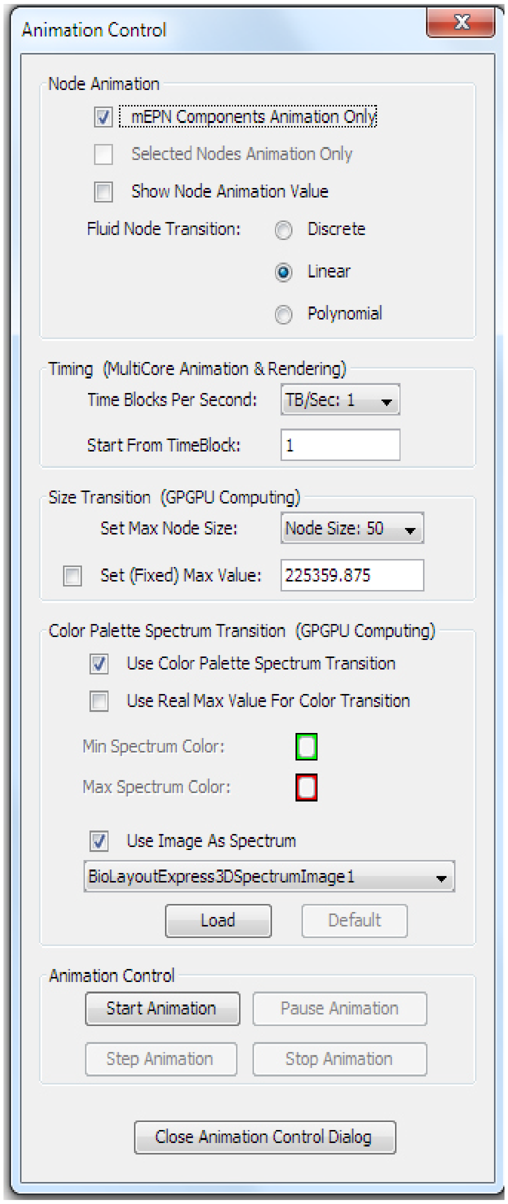
Animation Control
Node Animation
mEPN Components Animation Only
Selected Nodes Animation Only
Show Node Animation Value
Fluid Node Transition:
Discrete
Linear
Polynomial
Timing (MultiCore Animation & Rendering)
Time Blocks Per Second: TB/Sec: 1
Start From TimeBlock: 1
Size Transition (GPGPU Computing)
Set Max Node Size: Node Size: 50
Set (Fixed) Max Value: 225359.875
Color Palette Spectrum Transition (GPGPU Computing)
Use Color Palette Spectrum Transition
Use Real Max Value For Color Transition
Min Spectrum Color:
Max Spectrum Color:
Use Image As Spectrum
BioLayoutExpress3DSpectrumImage1
Load
Default
Animation Control
Start Animation
Pause Animation
Step Animation
Stop Animation
Close Animation Control Dialog
