# Supplementary figures and images for: Modelling the Structure and Dynamics of Biological Pathways
Source: PLoS Biol. 2016 Aug 10;14(8):e1002530. doi: 10.1371/journal.pbio.1002530 (PMC4980033; doi:10.1371/journal.pbio.1002530)

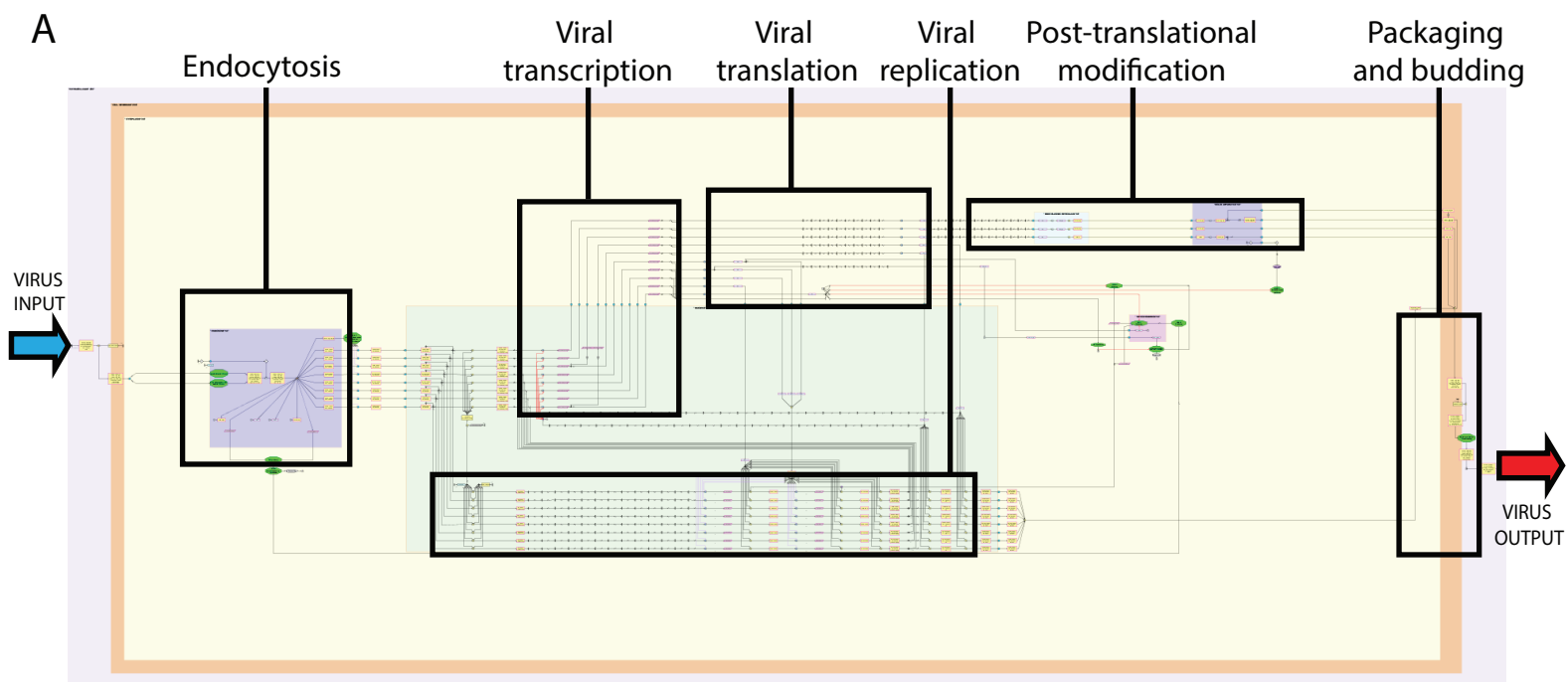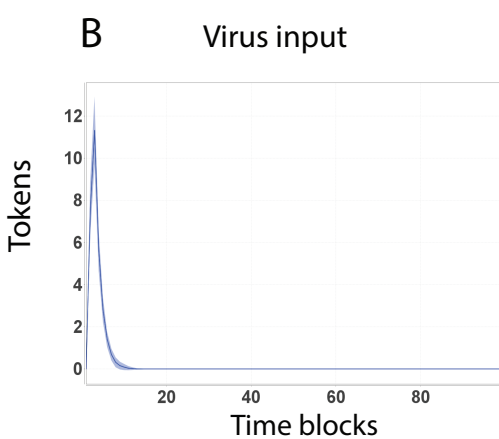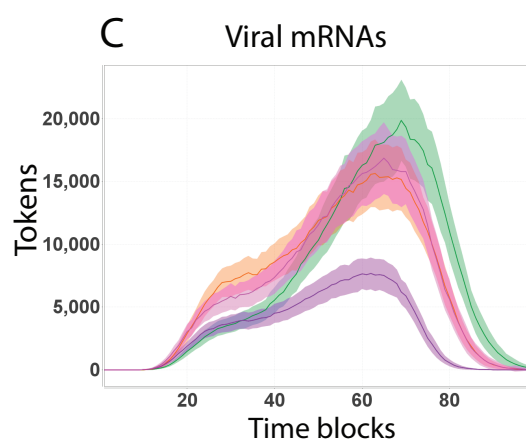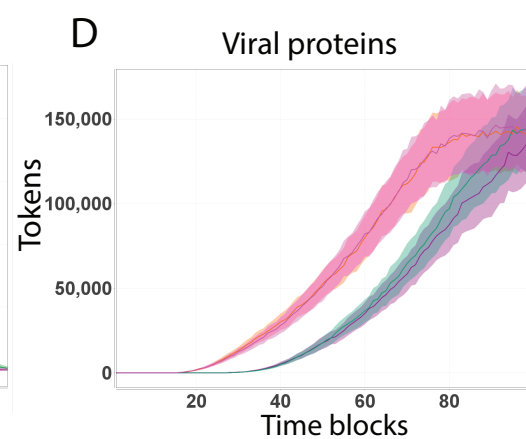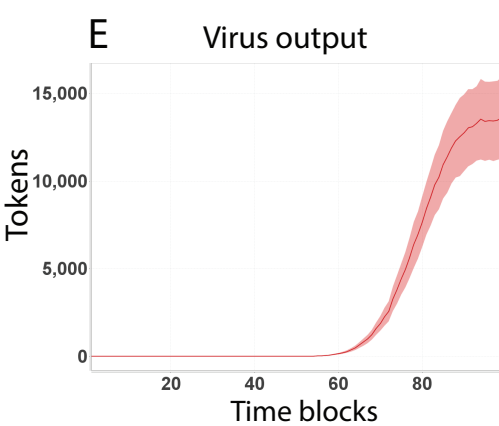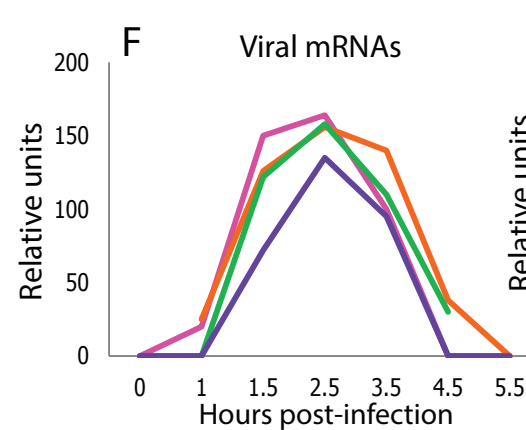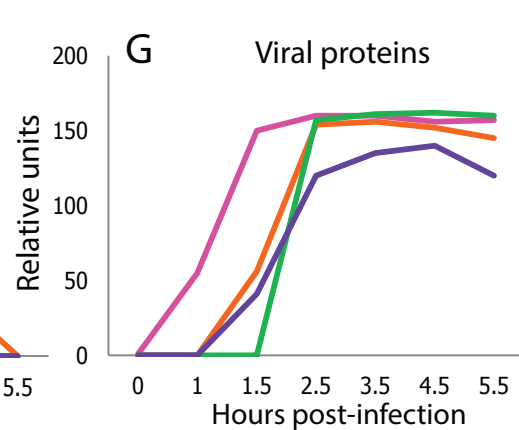

Supplement: S2 Fig — (A) Illustration of the modular structure of the IAV life cycle that facilitates its readability and allows for pathway expansion as new data become available or as a focus of interest grows. (B) When our virtual cell is challenged by a limited amount of virus (MOI 10 for two time blocks, ten virions are represented by ten input tokens, an accumulation of the structural components haemagglutinin (HA), neuraminidase (NA), matrix protein 1 (M1), and nonstructural protein 2 (NS2) both at the (C) mRNA and (D) protein level leading to (E) viral progeny (approximately 1E4 virions/cell Virus Output). The model outputs are comparable to the levels of (F) mRNA and (G) protein accumulation seen in previously reported experimental in vitro infection. (PDF) [file pbio.1002530.s002.pdf]
