# Supplementary material for: Modelling the Structure and Dynamics of Biological Pathways
Source: PLoS Biol. 2016 Aug 10;14(8):e1002530. doi: 10.1371/journal.pbio.1002530 (PMC4980033; doi:10.1371/journal.pbio.1002530)
Supplement: S1 Text — (DOCX) [file pbio.1002530.s006.docx]

**Modelling the structure and dynamics of biological pathways**

O’Hara *et al.*, S1 file

See also associated protocols paper (Livigni *et al.* BioRxiv <http://biorxiv.org/content/early/2016/04/04/047043>)

**Methods**

**Amendments to the modified Edinburgh Pathway Notation (mEPN) scheme**

The mEPN scheme represents our accumulated experience in building pathway models of complex biological systems. Our aim has been to develop a graphical language capable of capturing the current knowledge of these pathways and present the information in a human readable form. First published in 2008(1) and refined in 2010(2), its development has been driven by solving issues in the modelling of a wide variety of pathway systems. Examples of these are available at: [www.virtuallyimmune.com](http://www.virtuallyimmune.com). Here we describe version 2.0 of the mEPN scheme which is now designed not only as a formalized graphical language but also as a method for constructing computational models. The notation system is supported by the excellent freeware graph editor, yEd (yWorks, Tübingen, Germany; [www.yworks.com](http://www.yworks.com)).

A major advance in mEPN version 2.0 is the division of nodes used in the construction of models into one of two categories; places or transitions. Pathway components (e.g. genes, proteins, complexes, biochemicals) being designated as place nodes; processes (e.g. binding, dissociation, phosphorylation) being designated as transition nodes. In essence, places are generally something that exists in the physical world, whereas transitions generally represent interactions or events that link components. The significance of this will become apparent when describing the computational modelling system below. We have also created a number of new entity (place) and process (transition) nodes that are specifically designed for use in computational models: a spacer node (depicted as a small white circle) functions as a place and is used to maintain the bipartite graph structure where otherwise two transitions would be linked; black rectangular transition nodes (as used in conventional depictions of Petri nets) allow the marking of sites where tokens are added to the system (input transitions); and black diamond shaped transition nodes (distribution/spacer transitions) are used in the dispersion of tokens from a place or to maintain the bipartite structure of the network. Other than this, component nodes have been defined for depicting RNA species (grey parallelogram), and a number of new processes nodes including those to denote secretion, mutation, translation, unknown, as well as a number of process nodes that map on to BioPAX terms (modulation, conversion, transport with biochemical reaction)(3) have been added to the mEPN scheme. This last category of additions has not been used in construction of models but may be used in our visualization of BioPAX (.owl) files(4). We have also rationalized the depiction of edges and only six edge types now exist. Three of these all operate identically in the modelling environment: the interaction edge (standard arrow) is used for most interactions; catalysis edge (purple with open circle at end) is used in depicting enzymatic reactions and connects an enzyme to the process node defining the reaction; and the action potential edge (green with open circle and three lines at the end) used to depict interactions between neurons. We have also introduced a competitive inhibition edge (red with open diamond end) in addition to the standard depiction of an inhibitor edge (red with perpendicular bar at end) which functions as a non-competitive inhibitor in the modelling environment. The functional difference between these is described later. The sixth type of edge, the non-covalent interaction edge (black, non-directional), cannot be used in the computational modelling environment but may be useful in graphical depictions. It allows the depiction of two or more entities being bound together, yet still retaining their distinct identities e.g. complexes bound to DNA or large structures made of numerous components each with a specific role or position within the larger entity e.g. the components of the nuclear pore or centromere. Finally, diagrams may also include a third type of node, the compartment node. These are used to provide a background to diagrams depicting different sub-cellular compartments, cell types or even tissues. They are defined as such by placing the compartment name in parentheses e.g. *Cytoplasm*. They should not be connected to any other node by an edge and those listed are presented as examples are not as an exhaustive list, nor is the colour shown designed to be prescriptive. Compartments play no part in the computational modelling process but provide context to components and events. A summary of the mEPN version 2.0 glyphs and node annotations is summarised in Fig 1. Models drawn using the mEPN scheme, can be imported and visualized within BioLayout *Express*^3D^(5) and from here exported as SBGN models for visualization within tools such as VANTED(6). These graphical representations of a biological pathway map on to the basic concepts of a Petri net (comparisons of these four outputs are shown in Fig 3).

**Implementation of the Signalling Petri Net (SPN) algorithm, a brief description of its functions and modifications to it**

The original implementation of the SPN algorithm as described by Ruths *et al.*(7), was written in the Python programming language and incorporated into the tool PathwayOracle(8). However, PathwayOracle is not simple to install and set up, has limited network editing capabilities and does not support the visualization of pathways other than in the standard Petri net convention i.e. white circles for places and black rectangles for transitions. For these reasons, the SPN algorithm was refactored in Java and added into the BioLayout *Express*^3D^ (5) codebase. The underlying code was parallelized to optimize the speed of calculations and a graphical user interface (GUI) was constructed for ease of use. For a detailed description of the SPN algorithm and its merits as compared to other modelling approaches, see the original work describing this algorithm(7).

Described below are the general characteristics of Petri net models, our implementation of the SPN algorithm and the rules governing token flow through the networks.

1. A characteristic of Petri nets is that models must be drawn as bipartite graphs i.e. place->transition->place. All places, transitions and edges function identically. The only exception being inhibition edges which as described below differ in their mode of action to other edges.
2. Models are created in yEd graph editor and saved as GraphML files. Prior to performing a simulation a model needs to be parameterized through the placement of tokens on places at the beginnings of a network i.e. on places that have no parents. This is performed by placing a transition node upstream of such places and marking the edge between the two with the number of tokens to be assigned to that place using the edge description field in yEd. A place without tokens, or where token number is set to zero, can be considered not to exist, a potentially useful concept when wanting create cell-specific models where some components are not expressed, or virtual knockouts. Similarly, an increase in expression or activity can be indicated by increasing the number tokens on a given component node.
3. GraphML files are loaded into the tool BioLayout *Express*^3D^ and a parser translates the diagrams drawn using the mEPN scheme. On import the conditions for SPN simulation are set i.e. mode of stochasticity, number of runs and time blocks etc., these being defined upon loading of the model (S1A Fig) and results can either be saved (S1B Fig) to file or visualised (S1C Fig). In BioLayout models can be visualized in a 2D or 3D environment.
4. A simulation is made up of time blocks and runs. Firing steps are nested within a larger time scale are called time blocks, in which each transition is fired exactly once in a random order. A simulation may involve one or many runs, each run beginning at the same initial token marking and calculated for a specified number of time blocks. The result of a simulation is defined as the average accumulation of tokens across individual runs on a given place, and as such can be associated with a measure of variance. The more runs performed the more the average flow at each time point will approximate a deterministic result, and results will be reproducible across separate simulations.
5. During a simulation, tokens flow in the direction of the edges from place to place via transitions. In this context all edges are the same their apparent length having no bearing on the nature of flow. In a linear network consisting of a line of alternating places and transitions (Fig 4), each time a transition is fired tokens will ‘flow’ down the network, the more tokens added the more tokens will flow downstream (Fig 4a.i). With a constant input of tokens, token accumulation at places downstream from the first place will rise to a constant level and remain ‘steady’, the rate of tokens entering the place approximating those leaving; places close to the start of the line accumulating tokens faster than those further downstream (Fig 4a.ii). When tokens are added in for a fixed number of time blocks i.e. a pulsed input, a wave of flow is produced, the amplitude of the wave reducing and its wave length increasing at places downstream of the input (Fig 4a.iii). The steadiness of flow i.e. variation over time under steady state conditions, will depend on factors effecting the stochasticity of simulations, namely the number of runs performed and assumptions concerning the randomness of signal propagation.
6. In the original implementation of the SPN algorithm the number of tokens moved from an upstream to downstream place when a transition is fired is entirely random, between one and the maximum number of tokens available, the so called uniform distribution mode (Fig 4B). As part of our investigations into the influence of stochasticity (how random is signal flow through biological systems and what effect does the degree of stochasticity have on a systems behaviour), we implemented two other forms of stochastic flow. The first of these options we have called standard normal mode, where the number of tokens taken forward when a transition is fired is not entirely random, but chosen from a standard normal distribution around the average (50% of maximum available) i.e. flow is semi-random. In the second option, called the deterministic mode, exactly half the tokens available upstream are moved downstream when a transition is fired. Even when this option is used flow remains non-deterministic as the order in which transitions are fired is still random thereby adding variability to the output of individual simulation runs. Selection of one or other of these modes of action can be selected when setting up a simulation experiment (S1 Fig).
7. When transitions receive numerous inputs they function as rule-based regulators of token flow downstream of them. A basic rule is that when the number of tokens on input places is not equal, the number of tokens taken forward will be based on the input place with the least number of tokens e.g. if one input has 50 tokens and a second has a 100, only 50 will be taken forward (Fig 3C). The exact number is determined not only by the initial number of tokens but is also dependent on the randomness of token flow. So if both inputs have 100 tokens it is likely that the output will be slightly less than this number as on average, the probability is that one of the two inputs will be less than this number on any transition firing. In the case of multiple inputs into places, inputs are additive (Fig 4C, green).
8. There are two modes dictating how tokens are dealt with when flow through a transition is either blocked or restricted. In the original implementation of the SPN algorithm when there are two inputs into a transition and one input has more tokens than the other, tokens not used during firing are retained on the input place with more tokens. So if one input has 10 tokens and the other 100, 90 tokens will remain on the second (assuming no further input of tokens). In an adjustment to this rule we have implemented a ‘consumptive’ mode whereby tokens will be lost from inputs as if the transition was fully open and the limiting input was not restricting flow. This prevents an accumulation of token numbers at places receiving a constant input with no outputs, instead token levels remain relatively constant. Both options are available within our implementation of the SPN algorithm.
9. In instances where there are multiple outputs from a transition, the number of tokens on the upstream place will be mirrored by downstream places i.e. flow is preserved. Where outputs come direct from a place, the number of tokens on the upstream place will divided randomly amongst downstream places i.e. flow is diluted (Fig 4D, purple).
10. Inhibitor edges have a unique function. They must originate from a place (the inhibitor) and connect with a transition node i.e. the process that is to be inhibited (Fig 4E). They are unusual in that no tokens will flow down an inhibitor edge. In the original implementation of the SPN algorithm any tokens residing on an inhibitor would completely block flow through the target transition. That is to say one token on an inhibitor could block the flow of a 100 through the transition. This we have defined as non-competitive inhibition, and depicted in the style of the classic inhibitor edge (red with perpendicular bar at its end). However as this did not strike us particularly relevant to biological networks we introduced the non-competitive inhibitor edge (red with open diamond end)*.* Here the number of tokens residing on the inhibitor is subtracted from the number of tokens flowing through the transition. Thus if at a particular point an inhibitor node has 30 tokens and the input into the transition is 100, the flow past the inhibited transition would be 70 tokens. As no tokens are lost through inhibitor edges it has become standard practice to draw inhibitor places with an associated output transition (sink). Without this flow through a negative feedback loop is completely and irrevocably stopped as tokens accumulated on the inhibitor remain there blocking further flow through the target transition. An inhibitor with a sink effectively has a half-life and depending on the configuration of the feedback loop e.g. path length between input and inhibitor and type of inhibition (amongst other factors), the system may exhibit oscillatory activity (Fig 4E).

**Running simulations and visualisation of results**

Described above are the basic rules governing the SPN algorithm as applied to Petri nets. Parameterisation of pathway models takes several forms. Firstly, the topology of the model itself is a major determinant of its activity with assumptions about particular steps, inclusion of inhibitory reactions and the connectivity between places all influencing the result. The second major determinant of activity is the mark-up of tokens at the start of a simulation i.e. the placement of tokens on network inputs to define the initial state of the system. In this context tokens represent the amount or activity of a given protein or other biological component. When a simulation is ‘run’ the activity of the system and individual components within it are represented by the flow of tokens that are accumulated or dissipated from one molecule to another over time based on the connectivity and co-dependencies within model. During a run all the transitions in the network are fired in a random order once. With each firing of a transition the number of tokens moved from one place to another is dependent on the mode of stochasticity, but in each case averages out to approximately 50% of the number of tokens upstream. The firing sequence of a simulation is determined by a signalling event generator which fires each transition in a random order once during each run. The SPN algorithm modifies traditional Petri nets by replacing the movement of one token every transition firing, by the movement of a number of tokens randomly chosen between zero and the maximum number of tokens at the input transition. A ‘time block’ is defined as the firing of all transitions once. The concept of ‘the number of runs’ is used to simulate the signalling dynamics of a pathway, and determines how many times the simulation is repeated, the results of each run being averaged. The more runs in a simulation the closer the number of tokens moved will be to the absolute average.

The version of mEPN described here now defines all pathway-associated concepts as being either places or transitions and when models are loaded into BioLayout *Express*^3D^ they are interpreted as such. BioLayout *Express*^3D^ is a generic network visualization tool originally designed and still used for the analysis of high dimensional data, such as transcriptomics data(5) or indeed graphs from any source. As such it has been developed to analyse very large networks and given the often complex topology of these graphs, we chose to visualize them in a 3D environment where graph structure can be better explored(5). When models are loaded into the tool the 2D mEPN notation is translated into the 3D equivalents of their 2D shape (there are a few exceptions to this where more complex 3D shapes are used such as in the case with AND and OR logic nodes). For a summary of the mapping of these concepts from 2D to 3D glyphs see Fig 3. Standard Petri net model depictions will also load into the tool and can be ‘run’ if constructed in yEd and saved as a GraphML file.

mEPN models viewed within BioLayout have been designed to appear very similar in terms of the layout of nodes and edges (Fig 3). There is also an option to view compartments as 2D sheets or 3D ‘containers’ in which the models are embedded. Whilst the appearance of models in BioLayout is designed to be similar to their 2D yEd equivalents, the navigation of models in 3D is quite different as they can be visualised from different directions and orientations. If GraphML files contain any nodes that fit the criteria that define transitions, BioLayout recognises that the user may be attempting to run a signalling Petri net and is provided with menus that allow them to set the parameters for simulation e.g. number of time blocks and runs, stochasticity mode etc. Computation of simulations usually takes only seconds even for large models. Once calculations are complete, the results can be visualized in number of ways. Firstly, placing the cursor over a node (place nodes only) will show a plot the accumulation of tokens over time for that node. This allows rapid examination of outcomes across the model. The second and similar way to view results is to select a number or indeed all of the nodes of a model and view the results within the ‘Class viewer’ tool. Here the profiles of selected nodes are plotted on the same graph allowing the comparison of outcomes between nodes. The class viewer provides information on selected nodes, for example the node name and the classification of the component, supports the refinement of the selection by clicking on and off node check boxes, and allows the export of results as pictures (.png files). A final way to view results is through the dynamic visualisation of token flow. In this mode the animation of node size and colour is used to represent token accumulation. In order to achieve this we have constructed an interface that allows the user to control many aspects of the animation (S1C Fig). Here users are provided with options to set the number of time blocks visualized per second, the size of the nodes when they reach a set maximum number of tokens, what that maximum is (by default it is set to be the maximum number accumulating in any node during a simulation which may be so large as to obscure smaller values in other nodes), the colour palette used to help reinforce visually the change in token value, and an ability to stop and start the animation. This mode provides a powerful visual medium to appreciate token flow in large network diagrams and can be used to troubleshoot the innate challenges associated with model construction. Indeed, the general *modus operandi* when optimizing models is to construct them in yED, test the flow characteristics in BioLayout and to solve issues encountered by going back to yED diagram and retesting the model. In reality the movement between the two tools is quick and model testing and improvement can be a rapid process. A more complete description of the process of model construction and testing is given in the associated protocols paper (Livigni *et al.* BioRxiv http://biorxiv.org/content/early/2016/04/04/047043).

**Flow characteristics through simple pathway motifs**

The most simple pathway motif consists of an ordered series of linear interactions. Drawn as a Petri net it consists of alternating string of places and transitions (Fig 4A). Tokens are added to the beginning of the network, the more tokens added to the first place, the more tokens will accumulate at downstream places (Fig 4Ai). When the input of tokens remains constant throughout a simulation, an equilibrium point is reached where the number tokens entering a place approximates those leaving. The further a place is away from the input in terms of the number of place-transition intermediates, the longer it takes for the equilibrium to be reached (Fig 4Aii). When token input is pulsed, the wave of tokens is wider but never reaches the same amplitude at downstream places (Fig 4Aiii). This is an inherent property of the SPN algorithm that token flow from individual transitions is random, as is the order of transition firing i.e. it is a stochastic modelling system. In the original implementation of the SPN algorithm, when a transition is fired a random number of tokens between 0 and the number on the upstream place are passed on to a downstream place. Consequently if run number is low then the passage of tokens is highly variable across time blocks, as the number of tokens passed on is based on the average flow over multiple runs. When the run number is high, the tokens being passed on per time block approximates to 50%. Given the average answer to simulating completely random flow over many runs is approximately half, we introduced two new modes of running the algorithm. In the first, called the ‘deterministic’ mode, exactly 50% of the tokens upstream are passed to the downstream place when a transition is fired. In the second, the ‘standard normal’ mode, the number of tokens passed on is based on a normal distribution around this figure. In this way varying run number and the mode transition firing can greatly affect the uniformity of token flow down a linear series of interactions (Fig 4B). Knowing exactly what settings best simulate the probabilistic flow of information through a biological system is more difficult.

A second type of simple motif is where there is an interaction between components, whereby there is more than one place up or downstream of a transition or place (Fig 4C). Perhaps the most common of these motifs are interactions between pathway components, in which transitions represent a given process e.g. binding, catalysis etc. In such reactions both reacting species influence the outcome and the rules governing token flow are quite simple. The number of tokens taken forward will be approximately half those on the upstream place with the least number of tokens when a transition fires. So if one protein is present/active at half the level of another, it will be this entity that determines the rate of token flow downstream. If a place receives multiple inputs, token flow into that place is additive. When a network’s connectivity expands as in the case where one enzyme may have multiple substrates, a transcription factor binds to the promoter of multiple target genes or a molecular aggregate dissociates, this is viewed as a place leading to multiple outputs. If a place feeds into a single transition and each downstream place is fed from this, downstream places will receive approximately the same number of tokens i.e. flow is conserved but the number of tokens in the system has effectively been increased (Fig 4D). If however a place feeds into multiple separate transitions, flow to downstream places will be divided amongst them i.e. flow is diluted and the number of tokens in the system is preserved (Fig 4D). Ultimately these two options for distributing signal are based on different underlying assumptions and depending on which assumption is deemed to be correct it can greatly influence a model’s behaviour.

Perhaps the most interesting network motif is the negative feedback loop. Negative feedback loops have been found to operate over a wide variety of different biological systems at almost every level. For every activating biological pathway, there would appear to be one or often more negative regulatory systems and numerous modelling approaches have been suggested for these systems(9). They act as means to regulate a system’s activity and generally involve a downstream product of an activating cascade feeding back to inhibit an earlier step in the process. An inhibitory interaction is indicated in mEPN as a red line with either perpendicular bar end (non-competitive) or with open diamond end (competitive). Both types of inhibitory edges are drawn originating from a place to a transition where they inhibit token flow through that transition. They are unique in the fact that tokens are not lost from the inhibitor node via these edges. Therefore when an inhibitor is drawn with their only output being an inhibitory edge, tokens will accumulate on that inhibitor effectively closing the target transition irrevocably. For this reason inhibitors are generally drawn with an output sink, thereby simulating the concept of them having a half-life. The non-competitive inhibitor edge (Fig 4Ei) operates as described by Ruths *et al.* and when one or more tokens are present on the inhibitor, the target transition will be closed irrespective of flow through it. The second, competitive inhibitor edge (Fig 4Eii) was introduced by us and here the number of tokens on an inhibitor is subtracted from the number flowing through the target transition i.e. if there are 25 tokens residing on an inhibitor and 100 potentially passing through the transition targeted by it, only 75 will be passed on. The other major determinant of activity flow through negative feedback loops is the distance (number of places and transitions) between the input and inhibitor places. The longer this is the greater time tokens have to accumulate in the system and therefore when they reach the inhibitor the longer the target transition will remain ‘closed’, as tokens continue to flow into it even after the upstream flow has been blocked. This is illustrated in Fig 4E, where the effect of flow can be seen by varying the length of the feedback loop and the assumptions around the how the inhibitor operates. When the distance between the input and inhibitor is short (n=1) flow is essentially supressed, when longer (n=10) the system behaves like a damped oscillator and when long (n=50) only then can a fully oscillating system be realised. The behaviour of these network motifs can be further influenced by the model of stochasticity employed and by the number of feedbacks present. It is an interesting observation that the more feedback systems present, the more effective the feedback is i.e. the more it resembles a full oscillatory system, due to the increased probability that tokens will reside on the inhibitor at any one time. This observation may explain why many biological systems possess multiple feedback inhibitors acting at different levels in the system.

Token flow through pathway motifs provides outcomes that fit with the known characteristics of interactions between biological components i.e. probabilistic outcomes from random interactions between different molecular species. Rules associated with token flow through transitions also seem to fit simple basic concepts associated with biological interactions e.g. the amount of complex or product of an enzymatic reaction produced is dependent on the amount of reactants available. The competitive inhibitor mode introduced to the SPN framework by us also works as one might predict an inhibitor working *in vivo.*  However, there is great flexibility as how one might draw or parameterise a feedback loop and a broad range of outcomes are possible based on the topology of the system and assumptions made. Simply put, the basic concepts governing flow are intuitive and operate as one might expect a biological system to work. One of the truly liberating aspects of the modelling environment described here is how such motifs can be modelled with direct reference to knowledge of the system as described in the literature and models can be built on a large scale.

**References**

1. Raza S, Robertson KA, Lacaze PA, Page D, Enright AJ, Ghazal P, et al. A logic-based diagram of signalling pathways central to macrophage activation. BMC systems biology. 2008;2:36.

2. Freeman TC, Raza S, Theocharidis A, Ghazal P. The mEPN scheme: an intuitive and flexible graphical system for rendering biological pathways. BMC Systems Biology. 2010;4:65.

3. Demir E, Cary MP, Paley S, Fukuda K, Lemer C, Vastrik I, et al. The BioPAX community standard for pathway data sharing. Nature Biotechnology. 2010;28(9):935-42.

4. Wright DW, Angus T, Enright AJ, Freeman TC. Visualisation of BioPAX Networks using BioLayout Express3D. F1000Research. 2014;3:246.

5. Freeman TC, Goldovsky L, Brosch M, van Dongen S, Maziere P, Grocock RJ, et al. Construction, visualisation, and clustering of transcription networks from microarray expression data. PLoS Comput Biol. 2007;3(10):2032-42.

6. Junker BH, Klukas C, Schreiber F. VANTED: a system for advanced data analysis and visualization in the context of biological networks. BMC bioinformatics. 2006;7:109.

7. Ruths D, Muller M, Tseng JT, Nakhleh L, Ram PT. The signaling petri net-based simulator: a non-parametric strategy for characterizing the dynamics of cell-specific signaling networks. PLoS Comput Biol. 2008;4(2):e1000005.

8. Ruths D, Nakhleh L, Ram PT. Rapidly exploring structural and dynamic properties of signaling networks using PathwayOracle. BMC systems biology. 2008;2:76.

9. de Jong H. Modeling and simulation of genetic regulatory systems: a literature review. Journal of Computational Biology. 2002;9(1):67-103.
