# Supplementary material for: Modelling the Structure and Dynamics of Biological Pathways
Source: PLoS Biol. 2016 Aug 10;14(8):e1002530. doi: 10.1371/journal.pbio.1002530 (PMC4980033; doi:10.1371/journal.pbio.1002530)
Supplement: S2 Text — (DOCX) [file pbio.1002530.s007.docx]

**Construction and parameterisation of influenza A virus (IAV) model**

O’Hara *et al.*, S2 file

We have used the modified Edinburgh Pathway Notation (mEPN) scheme to compile two models of the IAV life cycle in human cells one in a cell e.g., epithelial cell that has no defence system (S2 Graphml), the other models the IAV life cycle in the context of a macrophage where host defece systems have been added to the model (S3 Graphml). To construct these models we drew upon the literature, and models already available in Reactome ([1](#_ENREF_1)) and elsewhere ([2](#_ENREF_2), [3](#_ENREF_3)). The layout of the models is based on a modular structure that facilitates readability and allows for pathway expansion. As well as functioning as pathway maps to be read as a summary of the literature, the models were also constructed to support computational modelling using the SPN Petri net algorithm.

For the most part initial testing and validation of the models was performed assuming that all host cell components depicted were present and in excess i.e., their initial loading was set 10,000 tokens. A basic check in constructing a large model such as this is to ensure that flow through the diagram is possible as it is frequently the case when model checking, to find issues where flow stops because the bipartite structure of the network has not been maintained or there are logic errors associated with its construction. The models as presented and all experiments shown are on based idea that the host cell is infected by a limited amount of virus i.e. multiplicity of infection (MOI) of 10 virus particles for 2 time blocks (10 virions are represented by 10 input tokens). Previously-published experimentally-derived dynamics of influenza infection were used to further parameterize the model to closely reproduce experimentally observed kinetics ([4](#_ENREF_4), [5](#_ENREF_5)). To obtain an output that correlated with experimental data we modelled one time block to be equivalent to approximately three minutes of an IAV infection and introduced delay motifs (linear strings of place-transition-place motifs) so that mRNA and proteins accumulated at the appropriate time points. In other areas of the diagram signal amplification markings were included to represent for instance, the multiplicity of transcripts generated when a gene is expressed.

All simulations were performed for 500 runs assuming the standard normal distribution mode for the token flow and simulations run over 100 time blocks (roughly equivalent to 5 h post-infection). When the virtual cell (S2 Graphml) is challenged by virus an accumulation of the structural components haemagglutinin (HA), neuraminidase (NA), matrix protein 1 (M1) and nonstructural protein 2 (NS2) both at the mRNA and protein level (S2C&D Fig) and viral progeny (~1E^4^ virions/cell Virus Output, S2E Fig) comparable to the levels of mRNA (S2F Fig) and protein (S2G Fig) accumulation seen in experimental in vitro infection ([2](#_ENREF_2)).

We then expanded the model (S3 Graphml) to generate a host-pathogen model of **influenza A virus infection** by combining the IAV life cycle model above with elements of our previously-published model of pathogen sensing and response pathways of macrophages ([6](#_ENREF_6)). In particular we focused on the effector pathways that neutralise viruses, with particular attention to inferferon-β signaling which plays an important role in innate immunity. This model is also available as an editable GraphML file and is shown in Figure 5A with components of the viral replication pathway highlighted in black and those from the macrophage host-defence pathway highlighted in pink, illustrating the modular structure of the pathway model.

An infection of human epithelial cells by IAV can produce over a 1,000 times more infectious viral particles than infection of monocytes and IAV infection of human alveolar macrophages with the H1N1 strain is abortive ([7](#_ENREF_7)). Furthermore, variants of certain host-defence molecules are known to lead to an increased susceptibility to influenza ([8](#_ENREF_8)). To evaluate the potential of SPN models to predict biological outcomes, we compared the outcome of a simulated IAV infection in our no-defence model (S2 Graphml), and two versions of the host-defence model (S3 Graphml) either resting or interferon-primed. Endogenous interferon-β is produced by the macrophage upon toll-like receptor stimulation. To simulate interferon-primed macrophage tokens were introduced on the IFNB1 protein so the interferon response was immediate rather than delayed. When loaded into BioLayout *Express*^3D^ (Figure 5B) and the SPN algorithm run, the model predicts that interferon-primed macrophages (pink) are more effective in aborting IAV infections resulting in greatly decreased virus output (Figure 5C) and increased apoptosis (Figure 5D) in comparison to epithelial cells (red) and non-primed macrophages (blue).

These models are provided as an example of the scale and complexity that can be realised using this modelling framework.

**References**

1. Joshi-Tope G, Gillespie M, Vastrik I, D'Eustachio P, Schmidt E, de Bono B *et al.* Reactome: a knowledgebase of biological pathways. Nucleic Acids Res 2005;33:D428-32.

2. Sidorenko Y, Reichl U. Structured model of influenza virus replication in MDCK cells. Biotechnol Bioeng 2004;88:1-14.

3. Matsuoka Y, Matsumae H, Katoh M, Eisfeld AJ, Neumann G, Hase T *et al.* A comprehensive map of the influenza A virus replication cycle. BMC systems biology 2013;7:97.

4. Hatada E, Hasegawa M, Mukaigawa J, Shimizu K, Fukuda R. Control of influenza virus gene expression: quantitative analysis of each viral RNA species in infected cells. J Biochem 1989;105:537-46.

5. Shapiro GI, Gurney T, Jr., Krug RM. Influenza virus gene expression: control mechanisms at early and late times of infection and nuclear-cytoplasmic transport of virus-specific RNAs. J Virol 1987;61:764-73.

6. Raza S, McDerment N, Lacaze PA, Robertson K, Watterson S, Chen Y *et al.* Construction of a large scale integrated map of macrophage pathogen recognition and effector systems. BMC systems biology 2010;4:63.

7. Short KR, Brooks AG, Reading PC, Londrigan SL. The fate of influenza A virus after infection of human macrophages and dendritic cells. J Gen Virol 2012;93:2315-25.

8. Everitt AR, Clare S, Pertel T, John SP, Wash RS, Smith SE *et al.* IFITM3 restricts the morbidity and mortality associated with influenza. Nature 2012;484:519-23.

9. Verhelst J, Parthoens E, Schepens B, Fiers W, Saelens X. Interferon-inducible protein Mx1 inhibits influenza virus by interfering with functional viral ribonucleoprotein complex assembly. J Virol 2012;86:13445-55.

10. Desai TM, Marin M, Chin CR, Savidis G, Brass AL, Melikyan GB. IFITM3 restricts influenza A virus entry by blocking the formation of fusion pores following virus-endosome hemifusion. PLoS Pathog 2014;10:e1004048.
